# Mortality and diagnostic practice variation in interstitial lung disease admissions: insights from a multicentre UK cohort study

**DOI:** 10.1136/bmjresp-2025-004017

**Published:** 2026-04-03

**Authors:** Laura Jane White, Jonathon Shaw, Bethan Powell, Nyan M Kyi, Alicia Sou, Gareth Edward Hughes, Dilanka Tilakaratne, Conal Hayton, Trishala Raj, Vi Truong, Nashwah Ismail, Nawat Khanijoun, Rebecca Huang, Emma Hardy, Zainab Aslam, Mahum Sohail, Mahzaib Babar, Amsal Amjad, Naayaab Khan, Martin Regan, Oby Okpala, Ragavilasini Suresh, Jerome Mcintosh, Amy Gadoud, Timothy Gatheral, Georges Ng Man Kwong

**Affiliations:** 1 Lancaster Medical School, Lancaster University, Lancaster, UK; 2 Respiratory Medicine, University Hospitals of Morecambe Bay NHS Foundation Trust, Kendal, UK; 3 Department of Respiratory Medicine, Stockport NHS Foundation Trust, Stockport, UK; 4 Department of Respiratory Medicine, Blackpool Teaching Hospitals NHS Foundation Trust, Blackpool, UK; 5 Department of Respiratory Medicine, Northern Care Alliance NHS Foundation Trust, Salford, UK; 6 Department of Respiratory Medicine, Bolton NHS Foundation Trust, Bolton, UK; 7 Division of Infection, Immunity & Respiratory Medicine, The University of Manchester, Manchester, UK; 8 Department of Respiratory Medicine, Manchester University NHS Foundation Trust, Wythenshawe Hospital, Manchester, UK; 9 School of Medical Sciences, The University of Manchester, Manchester, UK; 10 Manchester Royal Infirmary, Department of Respiratory Medicine, Manchester University NHS Foundation Trust, Manchester, UK; 11 Department of Respiratory Medicine, Tameside and Glossop Integrated Care NHS Foundation Trust, Ashton-under-Lyne, UK; 12 The Pears Cumbria School of Medicine, Imperial College London, Carlisle, UK

**Keywords:** Interstitial Fibrosis, Clinical Epidemiology, Palliative Care, Idiopathic Pulmonary Fibrosis, Imaging/CT MRI etc

## Abstract

**Background:**

Interstitial lung diseases (ILDs) are a heterogeneous group of often progressive, unpredictable diseases. They frequently result in hospitalisations secondary to respiratory decompensation, termed ILD-related admissions. A proportion are due to an acute exacerbation of ILD (AEILD). All are associated with high mortality but are poorly characterised in real-world populations.

**Aim:**

To evaluate mortality outcomes and associated risk factors following ILD-related hospital admissions, including AEILD.

**Methods:**

We conducted a multicentre retrospective cohort study of primary International Classification of Diseases Version 10 coded admissions for ILD between 1 January 2017 and 31 December 2019 across 11 NHS hospitals in the North West of England. AEILD events were classified using clinical criteria: <30-day respiratory deterioration not secondary to cardiac failure, pulmonary embolism or pneumothorax. The AEILD subgroup was divided into those with CT confirmation (definite AEILD) and without CT confirmation (suspected AEILD). Primary outcome was time from admission to death. Statistical analyses included Kaplan-Meier and multivariate proportional hazards modelling.

**Results:**

Of 938 ILD-related admissions, 54.5% met study AEILD criteria. Overall, cumulative all-cause mortality to 90-days post-discharge was 40.2%. For the AEILD cohort, cumulative all-cause mortality to 90-days post-discharge was 47.6%. Median survival of the AEILD cohort was 107 days (95% CI 87.0 to 141.0 days) and the other ILD-related admission cohort 241.0 days (95% CI 208.0 to 308.0 days), with a statistically significant difference in survival (p<0.0001). 37.6% (192/511) of AEILD events had CT confirmation. Within the AEILD subgroup, median survival was higher in the CT group (144 days vs 100 days, p=0.027). AEILD was independently associated with mortality in a multivariate model. Preadmission oxygen, age and neutrophilia were associated with mortality in both ILD-admission and AEILD 90-day all-cause mortality models. 13.9% of admissions had documented palliative care input.

**Conclusions:**

Mortality associated with ILD-related admissions is high, with AEILD events independently associated with mortality. Findings highlight the need for improved education, access to palliative care and targeted AEILD research.

**WHAT IS ALREADY KNOWN ON THIS TOPIC**

- Hospital admissions in interstitial lung disease (ILD) carry a high risk of mortality, particularly when precipitated by an acute exacerbation of ILD (AEILD). Prior international surveys have highlighted clinician heterogeneity in the approach to AEILD, but there is very limited real-world data describing admission outcomes, diagnostic and treatment patterns from the UK.

**WHAT THIS STUDY ADDS**

- This study adds to the understanding that AEILD conveys poor survival outcomes and highlights age, preadmission oxygen use and neutrophilia as poor prognostic indicators. It highlights underuse of CT for diagnostic confirmation and demonstrates that a lack of CT confirmation is associated with shorter survival in simple modelling. It also demonstrates low palliative care inpatient service utilisation.

**HOW THIS STUDY MIGHT AFFECT RESEARCH, PRACTICE OR POLICY**

- These findings highlight the urgent need for consistent diagnostic pathways, equitable access to CT imaging and early multidisciplinary input for AEILD. Improved education of the non-specialist, patients and their relatives could improve recognition and outcomes in this high-risk population—including timely access to palliative care and acute admission burden.

## Introduction

 Interstitial lung diseases (ILDs) represent a heterogeneous group of disorders affecting the lung parenchyma, causing a spectrum of disease from inflammation to fibrosis.^1^ The UK has the highest number of patients with ILD in Europe with an approximate incidence of 4.6–8.65 per 100 000 people per year.^2^ The disease trajectory is variable, but often results in hospitalisation secondary to respiratory decompensation. Hospital admission in ILD is associated with significant morbidity and mortality.^3 4^ A subset of admissions is secondary to an acute exacerbation of ILD (AEILD). AEILD is defined clinically by a less than 30-day deterioration in symptoms not secondary to thromboembolic, cardiac or pneumothorax events. Radiologically, they are defined via high-resolution CT (HRCT) demonstrating evidence of bilateral ground-glass changes superimposed on a chronic ILD.^5^ Both must be present to diagnose a definite AEILD event. If only the clinical criterion is met, a diagnosis of suspected AEILD is made.^6^ While AEILD was initially defined among a cohort of patients with idiopathic pulmonary fibrosis (IPF), it has been extrapolated more recently to encompass all ILDs. Retrospective data have estimated annual incidence of AEILD at 9%, with AEILD events accounting for an estimated 41% of ILD-related admissions.^7 8^

It is recognised that ILD-related hospital admissions have a significant impact on disease trajectory, with both high inpatient and 90-day mortality rates observed.^48^,^10^ However, there is limited worldwide data reporting ILD-related admission outcomes. Management of ILD-related hospitalisations and AEILDs is currently guided by international consensus statements based on expert opinion. These statements recommend supportive interventions, although the scope of recommendations is limited due to lack of robust evidence.^6 11^ At present, only one negative randomised control trial in the treatment of AEILD has been published^12^ and there is a major need to increase research capacity in AEILD.^13^ There is also recognised heterogeneity among the international community in diagnosis and management of AEILD events—including use of CT imaging. Europe has the lowest rates of CT use in AEILD diagnosis (67% vs 91% in Asia) but no UK-specific data are currently available.^14^

We aimed to understand the role of ILD-related hospitalisation in ILD all-cause mortality using real-world data. Secondary aims included understanding risk factors associated with 90-day all-cause mortality in ILD-related hospital admissions and AEILD, alongside diagnostic and management patterns in AEILD. By increasing our understanding from real-world data, we can hope to identify research and service design priorities.

## Methods

### Study setting and population

We performed a multicentre retrospective observational cohort study of ILD-related admissions in adults ≥18 years old using International Classification of Diseases Version 10 (ICD-10) coding across a 3-year period (1 January 2017 to 31 December 2019) from seven NHS trusts, encompassing 11 NHS secondary and tertiary hospitals, in the North West of England, covering an estimated population of 7.3 million people.^15^ ICD-10 codes of B22.1, D86.0, D86.2, J67.0–67.9, J70.2–70.4, J84.1, J84.8 and J84.9 within the primary diagnosis of admission event triggered inclusion in the study (online supplemental table 1). Admissions for attendance at day-case procedures, for provision of medications or lack of available medical records were excluded. Any prior documented dissent for use of data in research resulted in exclusion.

Medical records were retrospectively reviewed and manually searched between June 2024 and April 2025. The primary outcome was the number of days from the start of ILD-related admission to death.^16^

### Patient and public involvement

Rationale for developing this research question initially stemmed from the published top ten priorities for progressive pulmonary fibrosis, of which its stakeholders included patients living with PPF and their relatives and carers. Among the top 15 questions, two were related to acute exacerbations: (a) ‘How can acute deteriorations of PPF be predicted in patients with PPF?’ and (b) ‘What is the best management of acute deterioration in PPF?’.^17^

Following the development of the study protocol, the concept and planned design was presented to a group of expert patients, the Research Champions through Action for Pulmonary Fibrosis, prior to IRAS submission. They confirmed the acceptability of the proposed design and assisted with planning of a dissemination strategy, including radio and newsletter appearances following publication.

### Data collection and variables

All data were sourced from coding and manual searching of medical records. All admissions meeting inclusion criteria underwent manual review of medical notes. Where available, data collection on demographics and pre-admission diagnoses included: age, gender, postcode, ethnicity, smoking history, comorbidities, ILD diagnosis, preadmission antifibrotic use, preadmission oxygen use, preadmission lung function tests (including forced vital capacity (FVC)) and transfer factor of the lung for carbon monoxide (TLCO). Comorbidities were converted to a Charlson Comorbidity Index value.^18^ Full postcodes were converted to deprivation deciles (DD), as per the 2019 UK Government English Indices of Deprivation data.^19^ A DD of 1 represents the most deprived and 10 the least deprived.

An underlying ILD diagnosis was obtained from manual searching of medical records, using multidisciplinary team (MDT) documentation, radiology and clinic letters where available. During data collection, we identified a group of patients without an MDT-confirmed diagnosis, but in whom evidence of fibrotic ILD was present. This group was labelled ‘pulmonary fibrosis as a diagnostic label’, reflecting real-world access to ILD MDT diagnosis.

Mortality-relevant data included AEILD status, at-admission investigations (white cell count and differential, C reactive protein (CRP), chest X-ray findings), data on CT of the chest, echocardiogram and bronchoscopy use, and provision of antibiotics or steroids throughout admission. Data on ventilatory support given, specialist respiratory and palliative reviews were also obtained.

Patients were allocated to AEILD or ‘other’ ILD-related admission cohorts. AEILD status was determined by manual searching of medical records and investigations relating to the admission event. For the purposes of this study, we defined AEILD clinically only by a <30-day clinical deterioration, not secondary to cardiac failure, thromboembolic disease or pneumothorax event. Manual searching of the clinical narrative, biochemical investigations (negative pro-B natriuretic peptide and negative D-dimer, where available), radiological investigations (chest X-ray and CT pulmonary angiogram (CTPA), where available) and medical treatments determined diagnosis and subsequently AEILD or ‘other’ ILD-related admission status. If a patient met this criterion, their admission was labelled ‘AEILD’ and if not, was labelled as ‘other’ ILD-related admission. Other ILD-related admissions reflected admissions secondary to disease progression and other acute cardiorespiratory events, including pneumothorax and pulmonary embolism, although these were not extracted as discrete variables. A respiratory review was considered a review from any respiratory-specialist healthcare professional. A palliative review was considered a review from any palliative-specialist healthcare professional.

The primary outcome was number of days from admission start date to date of death.

### Statistical analysis

To power the study to achieve statistical significance, we used a predicted all-cause mortality of 60% in the follow-up period, a mortality-based model of ten predictors and an R-squared of 0.1, determining the inclusion of 850 admission events. We chose a multicentre, 3-year inclusion period to achieve this threshold.

All statistical analysis was undertaken using SPSS V.31.0 and Python V.3.9.7, with the ‘statsmodel’ package for Cox proportional hazards modelling. Continuous observational data were analysed by mean (SD). Categorical observational data were reported as frequencies (percentage). Admissions were grouped into AEILD or other primary admission based on clinical criterion defined above. For subanalysis of the AEILD cohort, patients were grouped by presence of CT-confirmed AEILD (definite AEILD) and absence of CT-confirmed AEILD (suspected AEILD).

Continuous data were assessed for normality using the Shapiro-Wilk test and compared using the unpaired t-test or Mann-Whitney U test dependent on presence of normal distribution. Categorical data were compared with the χ^2^ test or Fisher’s exact test if the expected count was less than five. Post hoc analysis of χ^2^ with multiple comparisons was undertaken using standardised residuals, converted to p values and compared with the Bonferroni correction for statistical significance.

For time-to-event outcomes, Kaplan-Meier survival analysis with log-rank comparison for two or more curves was undertaken. Mean and median survival were calculated. To quantify risk for all-cause mortality, HRs with corresponding 95% CIs were estimated using a multivariate Cox proportional hazards model using the ENTER method. Variables were selected and entered into the model simultaneously. Variable selection was informed by both clinical relevance and prior evidence, rather than data-driven stepwise methods, to minimise model overfitting. Demographic characteristics (eg, age, gender, ethnicity) were included to assess the impact of non-modifiable risk factors, while clinical and inflammatory variables were chosen for their known or hypothesised relevance to mortality risk in ILD populations.^3 20 21^ For the AEILD cohort subanalysis, CT confirmation status and treatment strategies were also included in the model. The proportional hazards assumption was checked using Schoenfeld residuals. Results yielding p<0.05 were considered statistically significant.

Complete case analysis was undertaken. Variables with high proportions of missing data (>15%), once data collection had been undertaken, were removed. FVC was originally planned to be included in the model but removed due to a high proportion of missing data (390/938 or 38.9%).

## Results

3451 admissions were identified. 2449/3451 (70.9%) of admissions were excluded. Main reason for exclusion was day case procedure (1984/2449, 81.1%, (online supplemental figure 1), with additional reasons including incorrect coding (67/2449), non-ILD related admissions (108/2449) and transplant-related attendances (158/2449). The remaining 1002 admission events all underwent manual searching of medical records and coding data. 64/1002 (6.4%) had insufficient data available to determine admission status, leaving 938 admissions for inclusion in analysis. Median follow-up among censored patients (those alive at the end of the study period) was 2125 days (range 1563–2968 days).

### Descriptive statistics

In total, 511/938 (54.5%) of admission events met study AEILD criteria and 427/938 (45.5%) were considered other ILD-related admissions.

Baseline and admission characteristics, including rates of CT confirmation, respiratory and palliative specialist review and treatments, for the overall dataset and AEILD subgroup are summarised in tables1  2, respectively.

**Table 1:** Summary of descriptive patient characteristics for overall, AEILD and other ILD-related admission cohorts

	Overall n=938	AEILD cohort n=511	Other cohort n=427	P value	Adjusted P value (Bonferroni)
Baseline characteristics					
Age (mean, SD)	74.18 (12.48)	73.87 (11.63)	74.55 (13.42)	0.058	
Gender–male (number, %)	521 (55.5)	288 (56.4)	233 (54.6)	0.582	
Ethnicity (number, %)				0.067	
Not stated	61 (6.5)	25 (4.9)	36 (8.4)	**0.029**	0.115
White	802 (85.5)	444 (86.9)	358 (83.8)	0.187	
Asian	68 (7.2)	40 (7.8)	28 (6.6)	0.455	
Black	7 (0.7)	2 (0.4)	5 (1.2)	0.167	
CCI (mean, SD)	4.46 (1.98)	4.38 (1.86)	4.54 (2.11)	0.165	
Deprivation decile (mean, SD)	4.62 (2.89)	4.62 (2.92)	4.63 (2.85)	0.980	
Ever-smoker (number, %)	488 (52.0)	262 (51.3)	226 (52.9)	0.786	
ILD subtype (number, %)				**<0.0001**	
Not stated	91 (9.7)	22 (4.3)	69 (16.2)	**<0.0001**	**<0.0001**
IPF	295 (31.4)	178 (34.8)	117 (27.4)	**0.015**	0.161
NSIP	84 (9.0)	52 (10.2)	32 (7.5)	0.152	
CTD-ILD	46 (4.9)	28 (5.5)	18 (4.2)	0.372	
HP	77 (8.2)	44 (8.6)	33 (7.7)	0.624	
Drug-related	22 (2.3)	9 (1.8)	13 (3.0)	0.196	
Industry-related	19 (2.0)	10 (2.0)	9 (2.1)	0.870	
Sarcoidosis	25 (2.7)	11 (2.2)	14 (3.3)	0.286	
PFD	221 (23.6)	126 (24.7)	95 (22.2)	0.387	
Unclassifiable	28 (3.0)	18 (3.5)	10 (2.3)	0.290	
Other	30 (3.2)	13 (2.5)	17 (4.0)	0.213	
On antifibrotics (number, %)	118 (12.6)	73 (14.3)	45 (10.5)	**0.001**	
Oxygen preadmission (number, %)				**0.013**	
Long-term	256 (27.3)	153 (29.9)	103 (24.1)	**0.046**	0.139
Ambulatory	78 (8.3)	50 (9.8)	28 (6.6)	0.075	0.224
None	604 (64.4)	308 (60.3)	296 (69.3)	**0.004**	**0.012**
FVC (L) (mean, SD)	2.11 (0.83)	2.16 (0.86)	2.03 (0.79)	0.102	
TLCO (mm Hg) (mean, SD)	3.88 (2.19)	3.37 (1.61)	4.64 (2.67)	**<0.0001**	
Admission characteristics					
Any CT of thorax performed (number, %)	326 (34.8)	192 (37.6)	134 (31.4)	0.05	
Bronchoscopy performed (number, %)	19 (2.0)	4 (0.8)	15 (3.5)	**0.0041**	
Respiratory specialist review (number, %)	508 (54.2)	350 (68.5)	158 (37.0)	**<0.0001**	
Palliative specialist review (number, %)	130 (13.9)	92 (18.0)	38 (8.9)	**0.0003**	
Steroids (number, %)				**<0.0001**	
Methylprednisolone					
Methylprednisolone 500 mg	14 (1.5)	14 (2.7)	0 (0.0)	**0.0006**	**0.002**
Methylprednisolone 1000 mg	23 (2.5)	20 (3.9)	3 (0.7)	**0.002**	**0.005**
None	862 (91.9)	460 (90.0)	402 (94.1)	**<0.0001**	**<0.0001**
Oral steroids				**<0.0001**	
Prednisolone >50 mg	19 (2.1)	14 (2.8)	5 (1.2)	0.087	0.523
Prednisolone 30–40 mg	390 (42.8)	282 (55.5)	108 (25.3)	**<0.0001**	**<0.0001**
Prednisolone 15–20 mg	51 (5.6)	33 (6.5)	18 (4.2)	0.126	
Prednisolone <10 mg	12 (1.3)	5 (1.0)	7 (1.6)	0.375	
None	422 (46.3)	153 (30.1)	268 (62.8)	**<0.0001**	**<0.0001**
Initial antibiotics (number, %)				**<0.0001**	
Intravenous	325 (34.6)	231 (45.2)	109 (25.5)	**<0.0001**	**<0.0001**
Oral	288 (30.7)	179 (35.0)	94 (22.0)	**0.002**	**0.007**
None	297 (31.7)	91 (17.8)	206 (48.2)	**<0.0001**	**<0.0001**
HFNO (number, %)	54 (5.8)	43 (8.4)	11 (2.6)	**0.0001**	
CPAP or NIV (number, %)	16 (1.7)	15 (2.9)	1 (0.2)	**0.002**	
Invasive ventilation (number, %)	2 (0.2)	0 (0.0)	2 (0.5)	0.607	

P value refers to comparison between AEILD and other cohorts using independent t-test or Mann-Whitney U test for continuous variables, and χ2 or Fisher’s exact test for categorical variables.

Post-hoc analysis of chi-squared was undertaken using standardised residuals, converted to P values and compared to the Bonferroni correction for statistical significance.

Results in bold indicate statistical significance (p value <0.05).

AE-ILD, acute exacerbation of ILD; CCI, Charlson Comorbidity Index; CPAP, continuous positive airway pressure; CTD-ILD, connective tissue disease-ILD; FVC, forced vital capacity; HFNO, high-flow nasal oxygen; HP, hypersensitivity pneumonitis; ILD, interstitial lung disease; IPF, idiopathic pulmonary fibrosis; NIV, non-invasive ventilation; NSIP, non-specific interstitial pneumonitis; PFD, primary fibrosis as diagnostic label; TLCO, transfer factor of the lung for carbon monoxide.

**Table 2:** Summary of descriptive patient characteristics for all AEILD, AEILD with CT confirmation (definite AEILD) and AEILD without CT confirmation (suspected AEILD)

	Overall AEILD cohort n=511	AEILD with CT (definite AEILD) n=192	AEILD without CT (suspected AEILD) n=319	P value	Adjusted P value (Bonferroni)
Baseline characteristics					
Age (mean, SD)	73.87 (11.63)	73.16 (12.83)	74.30 (10.84)	0.549	
Gender–male (number, %)	288 (56.4)	113 (58.9)	175 (54.9)	0.378	
Ethnicity (number, %)				0.312	
Not stated	25 (4.9)	13 (6.8)	12 (3.8)	0.127	
White	444 (86.9)	165 (85.9)	279 (87.5)	0.621	
Asian	40 (7.8)	14 (7.3)	26 (8.2)	0.726	
Black	2 (0.4)	0 (0.0)	2 (0.6)	0.271	
CCI (mean, SD)	4.38 (1.86)	4.17 (1.91)	4.51 (1.82)	0.094	
Deprivation decile (mean, SD)	4.62 (2.92)	4.82 (2.98)	4.50 (2.89)	0.225	
Ever-smoker (number, %)	262 (51.3)	109 (56.8)	153 (48.0)	0.134	
ILD subtype (number, %)				0.209	
Not stated	22 (4.3)	12 (6.2)	10 (3.1)	**0.019**	0.208
IPF	178 (34.8)	60 (31.2)	118 (37.0)	0.093	
NSIP	52 (10.2)	21 (10.9)	31 (9.7)	0.187	
CTD-ILD	28 (5.5)	12 (6.2)	16 (5.0)	0.659	
HP	44 (8.6)	20 (10.4)	24 (7.5)	0.553	
Drug-related	9 (1.8)	0 (0.0)	9 (2.8)	0.259	
Industry-related	10 (2.0)	5 (2.6)	5 (1.6)	0.413	
Sarcoidosis	11 (2.2)	6 (3.1)	5 (1.6)	0.240	
PFD	126 (24.7)	45 (23.4)	81 (25.4)	0.620	
Unclassifiable	18 (3.5)	6 (3.1)	12 (3.8)	0.705	
Other	13 (2.5)	5 (2.6)	8 (2.5)	0.947	
On antifibrotics (number, %)	73 (14.3)	21 (10.9)	52 (16.3)	0.240	
Oxygen preadmission (number, %)				**0.0004**	
Long-term	153 (29.9)	39 (20.3)	114 (35.7)	**0.0002**	**0.0005**
Ambulatory	50 (9.8)	17 (8.9)	33 (10.3)	0.583	0.999
None	308 (60.3)	136 (70.8)	172 (53.9)	**0.0002**	**0.0005**
FVC (L) (mean, SD)	2.16 (0.86)	2.20 (0.81)	2.14 (0.88)	0.304	
TLCO (mm Hg) (mean, SD)	3.37 (1.61)	3.46 (1.49)	3.31 (1.69)	0.210	
Admission characteristics					
Respiratory specialist review (number, %)	350 (68.5)	152 (79.2)	198 (62.1)	**0.0001**	
Palliative specialist review (number, %)	92 (18.0)	28 (14.6)	64 (20.1)	0.072	
Steroids (number, %)					
Methylprednisolone				**0.0096**	
Methylprednisolone 500 mg	14 (2.7)	9 (4.7)	5 (1.6)	**0.036**	0.109
Methylprednisolone 1000 mg	20 (3.9)	13 (6.8)	7 (2.2)	**0.0098**	**0.029**
None	460 (90.0)	164 (85.4)	296 (92.8)	**0.0071**	**0.021**
Oral steroids				**0.021**	
Prednisolone >50 mg	14 (2.8)	10 (5.2)	4 (1.3)	**0.008**	**0.048**
Prednisolone 30–40 mg	282 (55.5)	109 (57.1)	173 (54.6)	0.584	0.104
Prednisolone 15–20 mg	33 (6.5)	6 (3.1)	27 (8.5)	**0.017**	0.999
Prednisolone <10 mg	5 (1.0)	1 (0.5)	4 (1.3)	0.414	0.999
None	153 (30.1)	56 (29.3)	97 (30.6)	0.760	0.999
Initial antibiotics (number, %)				0.128	
Intravenous	231 (45.2)	91 (47.4)	140 (43.9)	0.440	
Oral	179 (35.0)	62 (32.3)	117 (36.7)	0.314	
None	91 (17.8)	32 (16.7)	59 (18.5)	0.601	
HFNO (number, %)	43 (8.4)	14 (7.3)	29 (9.1)	0.478	
CPAP or NIV (number, %)	15 (2.9)	3 (1.6)	12 (3.8)	0.153	
Invasive ventilation (number, %)	0 (0.0)	0 (0.0)	0 (0.0)	N/A	
Admission outcome					
Death during inpatient admission (number, %)	103 (20.2)	32 (16.7)	71 (22.3)		
Cumulative %	20.2	16.7	22.3		
Death within 30 days of discharge (number, %)	49 (9.6)	15 (7.8)	34 (10.7)		
Cumulative %	29.7	24.5	33		
Death within 90 days of discharge (number, %)	91 (17.8)	39 (20.3)	52 (16.3)		
Cumulative %	47.6	44.8	49.3		
Death >90 days from discharge (number, %)	239 (46.6)	93 (48.4)	145 (45.5)		
Cumulative %	94.1	93.2	94.8		
Alive (number, %)	30 (5.9)	13 (6.8)	17 (5.3)		
Cumulative %	100	100	100		

P value refers to comparison between AEILD and other cohorts using independent t-test or Mann-Whitney U test for continuous variables, and χ2 or Fisher’s exact test for categorical variables.

Post-hoc analysis of chi-squared was undertaken using standardised residuals, converted to P values and compared to the Bonferroni correction for statistical significance

Results in bold indicate statistical significance (p value <0.05).

AE-ILD, acute exacerbation of ILD; CCI, Charlson Comorbidity Index; CPAP, continuous positive airway pressure; CTD-ILD, connective tissue disease-ILD; FVC, forced vital capacity; HFNO, high-flow nasal oxygen; HP, hypersensitivity pneumonitis; ILD, interstitial lung disease; IPF, idiopathic pulmonary fibrosis; N/A, not available; NIV, non-invasive ventilation; NSIP, non-specific interstitial pneumonitis; PFD, primary fibrosis as diagnostic label; TLCO, transfer factor of the lung for carbon monoxide.

### Primary outcome: Mortality rate and time-to-event analysis

Overall cumulative all-cause mortality to 90-days post-discharge was 40.2% (377/938). For the AEILD cohort, cumulative all-cause mortality to 90-days post-discharge was 47.6% (243/511). For the other ILD-related admission cohort, cumulative all-cause mortality to 90-days post-discharge was 31.4% (134/427).

**Figure 1:**
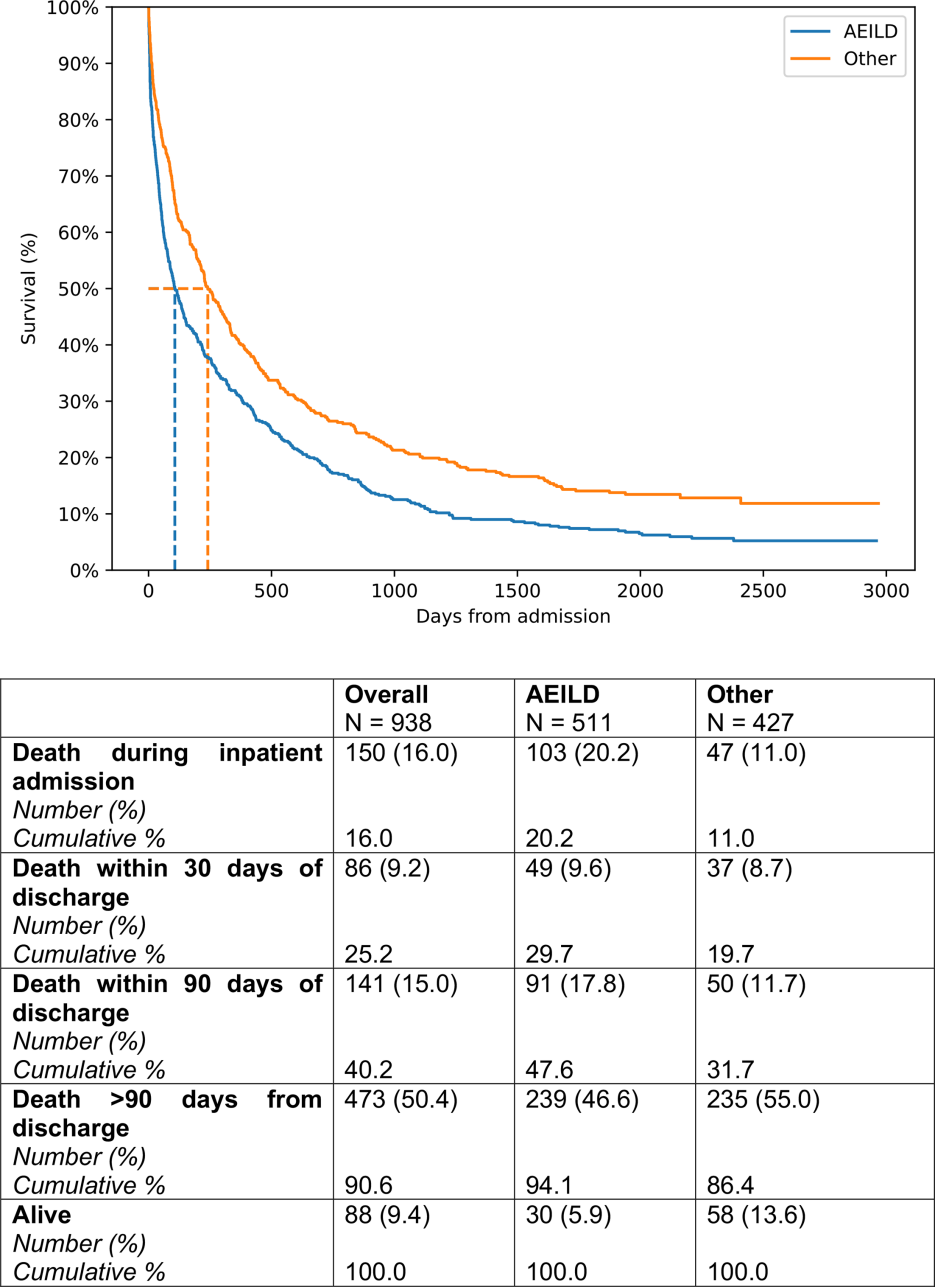
Kaplan-Meier analysis of interstitial lung disease-related admissions, comparing entire AEILD cohort (encompassing definite AEILD, with CT confirmation and suspected AEILD, with clinical definition only met) versus other ILD-related admissions. Median and mean survival of the AEILD cohort was 107 days (95% CI 87.0 to 141.0 days) and 408.6 days (95% CI 355.9 to 465.3 days), respectively. Median and mean survival of the other cohort was 241.0 days (95% CI 208.0 to 308.0 days) and 621.1 days (95% CI 549.5 to 700.3 days), respectively. There was a statistically significant difference in survival between the cohorts with log-rank comparison (p<0.0001). AEILD, acute exacerbations of interstitial lung disease; other, other interstitial lung disease-related admission reason.

Median and mean survival of the AEILD cohort was 107 days (95% CI 87.0 to 141.0 days) and 408.6 days (95% CI 355.9 to 465.3 days). Median and mean survival of the other cohort was 241.0 days (95% CI 208.0 to 308.0 days) and 621.1 days (95% CI 549.5 to 700.3 days). The difference in survival between the two groups was statistically significant (p<0.0001; figure 1).

### Secondary outcomes

Ten variables were included in a multivariate model for all-cause mortality across the ILD-related hospital admissions (table 3).

**Table 3:** Summary table of multivariate Cox regression analysis on 90-day all-cause mortality for all ILD-related admissions and AEILD subgroup analysis

	90-day all-cause mortality for ILD-related admissions multivariate HR	Lower 95% CI	Upper 95% CI	P value	90-day all-cause mortality in AEILD multivariate HR	Lower 95% CI	Upper 95% CI	P value
Age	1.032	1.017	1.046	**<0.0001**	1.04	1.019	1.061	**0.0001**
Gender								
Female	Reference	Reference	Reference		Reference	Reference	Reference	
Male	1.35	1.072	1.7	**0.011**	1.126	0.833	1.524	0.440
Ethnicity								
White	Reference	Reference	Reference					
Asian	0.589	0.357	0.97	**0.038**				
Not stated	1.66	1.091	2.523	**0.018**	N/A	N/A	N/A	N/A
CCI	1.010	0.938	1.087	0.794	0.95	0.852	1.059	0.357
ILD subtype								
Not known	Reference	Reference	Reference		Reference	Reference	Reference	
IPF	1.039	0.656	1.644	0.871	1.035	0.499	2.144	0.927
NSIP	0.557	0.207	1.496	0.246	0.491	0.101	2.378	0.377
CTD-ILD	1.278	0.748	2.185	0.370	1.056	0.471	2.366	0.895
HP	0.713	0.358	1.418	0.335	0.575	0.219	1.507	0.260
Drug-related	0.242	0.114	0.517	**0.0002**	0.134	0.044	0.411	**0.0004**
Industrial (asbestosis, silicosis)	0.776	0.293	2.057	0.610	0.826	0.216	3.154	0.780
Sarcoidosis								
PFD	0.471	0.161	1.379	0.169	0.171	0.021	1.369	0.096
Unclassifiable	0.899	0.357	2.265	0.821	1.103	0.355	3.425	0.866
Other	0.802	0.495	1.301	0.372	0.548	0.258	1.166	0.118
	1.106	0.541	2.261	0.782	0.528	0.167	1.671	0.277
Antifibrotic use								
None								
Nintedanib	N/A	N/A	N/A	N/A	Reference	Reference	Reference	
Pirfenidone					0.877	0.451	1.706	0.700
					0.977	0.595	1.606	0.927
Oxygen status								
None	Reference	Reference	Reference		Reference	Reference	Reference	
Long-term	1.965	1.548	2.495	**<0.0001**	1.853	1.324	2.594	**0.0003**
Ambulatory	1.384	0.933	2.053	0.107	1.336	0.798	2.236	0.271
AEILD status								
Other								
AEILD	Reference				N/A	N/A	N/A	N/A
	1.490	1.183	1.877	**<0.0007**				
Neutrophil count	1.077	1.045	1.11	**<0.0001**	1.084	1.039	1.131	**0.0002**
Monocyte count	0.889	0.69	1.144	0.360	0.796	0.55	1.153	0.228
CRP	1.003	1.001	1.004	**0.002**	1.002	1	1.005	**0.040**
CT thorax performed								
No	N/A	N/A	N/A	N/A	Reference	Reference	Reference	
Yes					0.847	0.608	1.178	0.324
Methylprednisolone								
No	N/A	N/A	N/A	N/A	Reference	Reference	Reference	
500 mg					2.095	0.984	4.459	0.055
1000 mg					1.682	0.789	3.585	0.178
Oral steroids								
No	N/A	N/A	N/A	N/A	Reference	Reference	Reference	
10 mg/day or less					0.882	0.189	4.117	0.874
15–20 mg/day					0.632	0.307	1.302	0.2140
30–40 mg/day					1.087	0.787	1.501	0.611
50 mg/day or more					1.178	0.458	3.028	0.733
Respiratory review								
No	N/A	N/A	N/A	N/A	Reference	Reference	Reference	
Yes					0.85	0.59	1.224	0.382

Results in bold indicate statistical significance (p value <0.05).

AEILD, acute exacerbation of ILD; CCI, Charlson Comorbidity Index; CRP, C reactive protein; CTD-ILD, connective tissue disease-ILD; HP, hypersensitivity pneumonitis; ILD, interstitial lung disease; IPF, idiopathic pulmonary fibrosis; N/A, not available; NSIP, non-specific interstitial pneumonitis; PFD, primary fibrosis as diagnostic label.

**Figure 2:**
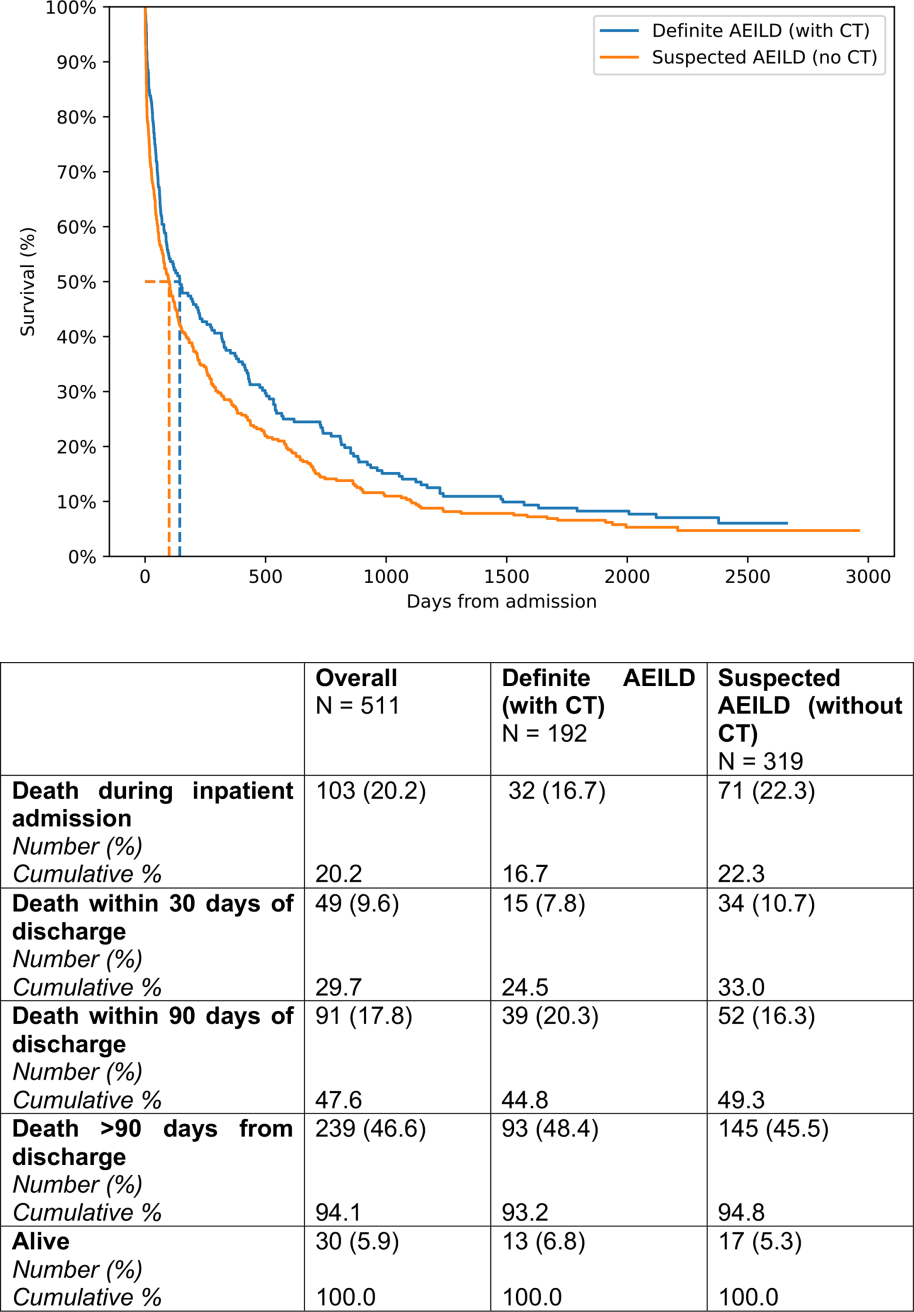
Kaplan-Meier analysis of AEILD cohort, comparing definite AEILD (with CT confirmation) versus suspected AEILD (without CT confirmation) using log-rank analysis. Mean and median survival of AEILD with CT was 476.8 days (95% CI 369.0 to 566.1) and 144.0 days (95% CI 87.0 to 237.0). Mean and median survival of AEILD without CT was 359.3 days (95% CI 289.1 to 425.2) and 100.0 days (95% CI 71.0 to 132.0), with a statistically significant difference between the two cohorts (p=0.027). AEILD, acute exacerbations of interstitial lung disease.

Subanalysis of the AEILD cohort was undertaken through assigning patients to definite or suspected AEILD by presence of CT imaging confirmation. 37.6% (192/511) of AEILD admissions had CT thorax imaging to confirm diagnosis, of which 90/192 (46.9%) were CTPA. Median and mean survival of AEILD with CT confirmation was 144.0 days (95% CI 87.0 to 237.0) and 476.8 days (95% CI 369.0 to 566.1). Median and mean survival of AEILD without CT confirmation was 100.0 days (95% CI 71.0 to 132.0) and 359.3 days (95% 289.1 days—425.2 days), with a statistically significant difference between the two cohorts (p=0.027; figure 2). Multivariate modelling of 90-day all-cause mortality for the AEILD sub-group was undertaken with 13 variables (table 3).

## Discussion

### Summary of findings

To the best of our knowledge, this is the largest real-world UK dataset analysing ILD-related admission outcomes. Our data demonstrates significant 90-day all-cause mortality following ILD-related admissions (40.2%). Mortality is significantly greater in an AEILD admission compared with other ILD-related admission reasons (median survival 107 days vs 241 days; p<0.0001). AEILD remained a risk factor for mortality in a multivariate model of 90-day mortality (HR 1.490, 95% CI 1.183 to 1.877; p=0.0007), alongside preadmission long-term oxygen use (HR 1.965, 95% CI 1.548 to 2.495; p<0.0001), age (HR 1.032, 95% CI 1.017 to 1.046; p<0.0001), male gender (HR 1.350, 95% CI 1.072 to 1.700; p=0.011) and neutrophilia (HR 1.077, 95% CI 1.045 to 1.110; p<0.0001). Ethnicity demonstrated a variable association, with Asian ethnicity (compared with a reference of White ethnicity) demonstrating protection against mortality (HR 0.589, 95% CI 0.357 to 0.970; p=0.038). Drug-related ILD was also associated with protection against mortality (HR 0.242, 95% CI 0.114 to 0.517; p=0.0002).

Given our AEILD cohort encompassed both definite AEILD and suspected AEILD, subanalysis was undertaken to understand the impact of CT confirmation on outcomes. Our data demonstrates a statistically significant difference in mortality between those with and without CT confirmation (median survival 144 days vs 100 days; p=0.027). Interestingly, it was our suspected AEILD cohort who experienced higher mortality. When taken into a multivariate model for all AEILD admissions, CT confirmation was not significantly associated with reduced risk of mortality (HR 0.847, 95% CI 0.608 to 1.178; p=0.324). Age, preadmission oxygen and neutrophilia were again associated with increased risk of mortality, and drug-related ILD reduced risk. Methylprednisolone and high-dose steroid use (>50 mg/day) demonstrated HRs suggesting increased risk of mortality within the model—but did not meet statistically significant thresholds.

These findings underscore the high mortality burden associated with ILD-related admissions and highlight diagnostic and management heterogeneity that may contribute to outcome variation.

### Study strengths and limitations

The main strength of this study is its multicentre design, encompassing several geographical regions—enhancing its power. By including secondary and tertiary hospitals, these combined strengths increase generalisability—but key limitations remain.

Our retrospective data is from 2017 to 2019. While this time frame was chosen to remove the confounding variable of the COVID-19 pandemic, it is important to recognise the change in treatment landscape since this timeframe, such as wider antifibrotic use—meaning the study may not reflect current patient outcomes.

The retrospective design also introduces the potential for recall and misclassification bias. This was particularly important for determining AEILD based on clinical, biochemical and radiological information available. While manually searching notes and investigations aimed to mitigate misclassification bias, inter-rater reliability was not assessed. Full CT reports nor discrete reasons for other ILD-related admissions were recorded—which may affect consistency in classification. Future studies should look to include both. Alongside this, cases were identified through ICD-10 coding. At present, no specific ICD-10 code exists for AEILD. This meant identification was reliant on the ILD diagnosis being coded as the primary reason for admission. Given the potential for incorrect coding, it is unlikely all cases during this time period have been identified. Manual searching of the clinical notes aimed to mitigate the inclusion of incorrectly coded admission events, but this raises the important need for an AEILD ICD-10 code, or equivalent, to help identify such cases for real world evidence review.

Missing data are also a further challenge of retrospective data. We observed high rates of this when recording lung function testing (FVC and TLCO), resulting in the need to remove FVC from our multivariate model. Additionally, hospital-only based data collection risks hospitalisation bias. Different results may be observed if respiratory decompensation events relating to ILD that do not result in hospital admission are also accounted for.

Despite covering a large geographic area in the North West of England, this study is region-specific and may not generalise to different health systems or ILD pathways observed outside of it. Further studies on a national scale, ideally with prospective data collection, are required to validate the results observed here.

### The role of AEILD in ILD admission mortality

AEILD events are not infrequent in the hospitalised ILD population. A prior retrospective study identifying AEILD events with clinical criterion only reported an AEILD rate of 52%^22^—similar to our observation. In studies where radiological and clinical criteria were used, fulfilling ‘definite AEILD’ criteria,^6^ the rate of AEILD in hospitalised patients was 20.8%–41%.^8 23^

AEILDs are frequent events and are associated with high mortality. Prior literature reports mortality of up to 100% in the intensive care setting.^24^ Our findings reinforce this high mortality among a UK population: AEILD admissions were associated with significantly higher 90-day mortality compared with other ILD-related admissions. Notably, median survival following AEILD admission was only 107 days and remained significantly different to other ILD-related admission reasons—even after multivariate adjustment. This suggests an AEILD event itself represents a distinct and high-risk clinical picture. However, there are a proportion of patients who survive beyond the 90-day mark for considerable periods (figure 1), raising the possibility of different phenotypes, causes and severities within the umbrella term AEILD. The possibility of differing AEILD severities was addressed in a post hoc analysis for exacerbators within the INPULSIS trial, with those meeting criteria for a serious adverse event demonstrating worse outcomes.^25^

The hypothesis of differing AEILD phenotypes, causes and severities is supported by the observed differences in AEILD outcomes between differing underlying ILD disease types,^26^ which is further suggested in our multivariate model—although not to a statistically significant threshold. The small numbers within each ILD sub-group may have impacted the ability to reach such thresholds. It is likely also limited by the choice to group by disease, rather than radiological pattern. The 2025 joint European Respiratory Society and American Thoracic Society guidelines have moved initial diagnostic labelling to a two-step process, first based on morphological patterns in pathology and imaging.^27^ This distinction appears important in AEILD, too, with usual interstitial pneumonia and nonspecific interstitial pneumonitis radiological patterns shown to have different survival outcomes.^28 29^ We did not collect radiological pattern data in this study. Future studies should look to categorising by radiological pattern, rather than disease, to understand the impact on prognosis more clearly.

The mechanisms driving poor outcomes in AEILD are poorly understood,^5^ and treatment options remain limited.^11 13^ The current international consensus guidance recommends supportive care but lacks specific pharmacological and non-pharmacological strategies.^6^ Our results support the argument that AEILD represents not only an acute crisis but also a turning point in the disease course. The elevated mortality risk underscores the need for early identification, prompt multidisciplinary input and targeted therapeutic trials alongside clear, defined, palliative and end-of-life care strategies.

### Impact of CT confirmation on AEILD outcomes

CT thorax imaging currently remains an essential part of the diagnostic criteria for confirming an AEILD.^6^ However, there is heterogeneity among its use internationally^14^—and patterns of its use have never been studied in a specific UK dataset.

In our real-world dataset, we demonstrate poor uptake of CT imaging to confirm an AEILD. And, even when undertaken, the majority are CTPA—increasing the challenge of accurately demonstrating the hallmark diffuse alveolar damage in AEILD given administration of contrast. Despite this, we still observed a significant difference in survival, whereby mortality risk increased if CT confirmation was not undertaken (suspected AEILD) in Kaplan-Meier survival analysis. There are some potential explanations for this. First, patients with AEILD often develop profound respiratory failure^30^—meaning they may not be safe to transfer to CT imaging to confirm the diagnosis. However, these data did not demonstrate significant differences in high-flow nasal oxygen use, non-invasive ventilation or specialist palliative care inpatient review. A second possibility is that CT is a marker for more intensive therapies and specialist care. The group with CT did demonstrate a higher respiratory review rate (62.1% in suspected AEILD vs 79.2% in definite AEILD; p=0.0001; table 2) and higher rate of methylprednisolone (1.6% in suspected AEILD vs 4.7% in definite AEILD for 500 mg; p=0.036; 2.2% in suspected AEILD vs 6.8% in definite AEILD for 1000 mg; p=0.0098; table 2) or high-dose (>50 mg/day) prednisolone use (1.3% in suspected AEILD vs 5.2% in definite AEILD, p=0.008; table 2). However, in a multivariate model of 90-day AEILD all-cause mortality, neither CT, nor respiratory review, nor steroid use met statistically significant thresholds for association with or protection from mortality. In fact, high-dose steroid use had numerical HRs associated with increased mortality (table 3)—but this may be confounded by the fact that the most unwell patients receive this therapy.^31^ Instead, increased age, preadmission long-term oxygen therapy and neutrophilia were significantly associated with increased all-cause mortality across AEILD admissions. Finally, a further consideration is that the suspected AEILD cohort has potential for significant heterogeneity, with the worse mortality outcomes observed being non-respiratory related. While ground glass opacities are not a specific pathological marker—and still have potential for misinterpretation—they must be taken in the context of clinical and biochemical markers to draw diagnostic conclusions. Future suspected and definite AEILD comparisons should consider interrogation of death certificate data combined with inter-relatability assessments of cohort classifications.

There are limited data comparing suspected versus definite AEILD outcomes. Post hoc analysis from the STEP-IPF trial found no significant difference in mortality between suspected and definite AE-IPF events—suggesting both convey significant postevent mortality.^32^ Our findings support this, with a multivariate model highlighting the complex interaction of many variables contributing to AEILD mortality.

These findings have important service and education implications. They emphasise the need for harmonised diagnostic criteria and greater accessibility of HRCT in the acutely unwell population, to ensure consistent identification and management of AEILD events. If CT remains an essential part of the diagnostic criteria, then low rates of CT use also highlight an essential education need for the non-respiratory physician who may see these patients in the emergency or acute medical take setting. They also raise the question of the possible need for a pragmatic, real-world diagnostic algorithm to standardise care—but these findings first need confirmation in a validatory cohort.

### Palliative care input

Our study highlights a concerning underutilisation of specialist palliative care services in a population with exceptionally high short and long-term mortality—greater than many common cancers. Less than one in five patients experiencing an AEILD received inpatient specialist palliative care reviews despite a 90-day mortality of just under 50%. There have been several calls to improve access and use of palliative care services in ILD, with a growing understanding of the unmet needs of this population.^33^,^35^ But a lower referral rate may reflect the clinical uncertainty ILD brings and the lack of direct palliative recommendations in the context of a respiratory decompensation or AEILD event.^6 36^ This reflects the lack of evidence in this area, and much of what is available is of low quality to base recommendations on.^6^ There is a well-recognised international heterogeneity in approaches to AEILDs, with 50%–71% of specialist clinicians reporting they routinely offer palliative care support in the context of ILD.^14^

In the UK, the National Institute for Clinical Excellence care quality standard 5 for IPF states: “people with IPF and their families and carers have access to services that meet their palliative care needs”.^37^ The recent British Thoracic Society ILD registry report summarising 2024 data demonstrates the heterogeneity in meeting this care standard across UK hospitals,^38^ further supported by our findings. However, our study may underappreciate the palliative care provided by respiratory and general physicians. Our study recorded palliative care input as a documented inpatient review by a palliative care specialist only. More recent palliative-focused studies have shown the merits of an MDT model to improve patient-reported outcomes.^39^ Follow-up studies should acknowledge palliative care input from the non-palliative specialist to build a full picture of the palliative care approaches in ILD admissions.

This current observed underutilisation of palliative care highlights an area of education for the non-specialist. Improved education and awareness of the poor prognosis, alongside improved diagnostic confirmation, may help to improve patient and carer access to the vital palliative and supportive care services they need.

## Conclusions

This retrospective study demonstrates high mortality in the 90 days following ILD-related inpatient admissions, especially in the context of AEILD. It demonstrates the real-world application of the AEILD diagnostic criteria, highlighting essential need for education among the non-ILD specialists to improve rates of CT use and timely palliative care utilisation.

## Supplementary material

10.1136/bmjresp-2025-004017 online supplemental file 1

## Data Availability

Data are available on reasonable request.
