# Supplementary material for: Mortality and diagnostic practice variation in interstitial lung disease admissions: insights from a multicentre UK cohort study
Source: BMJ Open Respir Res. 2026 Apr 3;13(1):e004017. doi: 10.1136/bmjresp-2025-004017 (PMC13052612; doi:10.1136/bmjresp-2025-004017)
Supplement: online supplemental file 1 [file bmjresp-13-1-s001.pdf]

Supplementary Materials

ICD-10 Code	Diagnosis
B22.1	HIV disease resulting in pneumocystosis
D86.0	Sarcoidosis of lung
D86.2	Sarcoidosis of lung and lymph node
J67.0	Farmer's lung
J67.1	Bagassosis
J67.2	Bird Fancier's lung
J67.3	Suberosis
J67.4	Malt Worker's lung
J67.5	Mushroom Worker's lung
J67.6	Maple bark-stripper's lung
J67.7	Air-conditioner and humidifier lung
J67.8	Other hypersensitivity pneumonitis due to organic dusts
J67.9	Hypersensitivity pneumonitis, unspecified
J70.2	Acute drug-induced interstitial lung disorders
J70.3	Chronic drug-induced interstitial lung disorders
J70.4	Drug-induced interstitial lung disorders, unspecified
J84.1	Other interstitial pulmonary diseases with fibrosis
J84.8	Other specified interstitial pulmonary diseases
J84.9	Interstitial pulmonary disease, unspecified

Supplementary Table 1: ICD-10 codes with full diagnostic names used to identify admissions for inclusion within the study.

CONSORT

TRANSPARENT REPORTING of TRIALS

CONSORT 2010 Flow Diagram

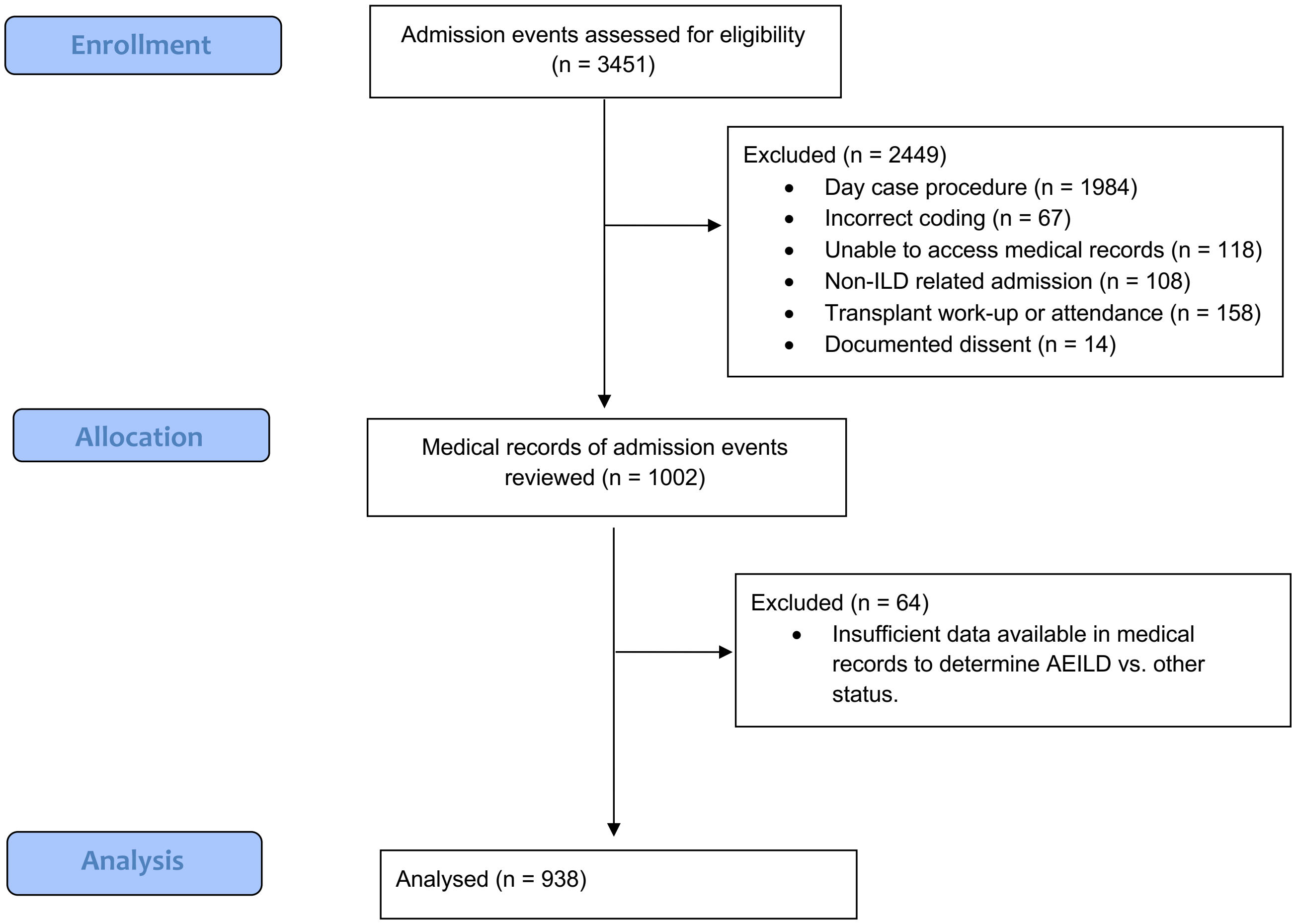

Supplementary Figure 1: CONSORT study flow diagram summarising records identified, reasons for exclusion and number of records included in analysis.

Abbreviations: ILD – interstitial lung disease; AE-ILD - acute exacerbation of interstitial lung disease; ICD-10 – international classification of diseases version 10.
